# High fat meals increases postprandial fat oxidation rate but not postprandial lipemia

**DOI:** 10.1186/s12944-019-1129-x

**Published:** 2019-10-23

**Authors:** Chih-Hui Chiu, Tsung-Jen Yang, Che-Hsiu Chen, Ming-Jing Zeng

**Affiliations:** 1 Department of Exercise Health Science, National Taiwan University of Sport, No.16, Sec. 1, Shuang-Shih Rd, Taichung, 404 Taiwan; 2 Department of Physical Education, National Taiwan Normal University, Taipei, 106 Taiwan; 3 Department of Sport Performance, National Taiwan University of Sport, Taichung, 404 Taiwan

**Keywords:** Intramuscular triglycerides, Insulin sensitivity, Exercise

## Abstract

**Background:**

This study investigated the effects of ingesting meals with the same calorie intake but distinct nutritional contents after exercise on postprandial lipemia the next day.

**Methods:**

Eight healthy male participants completed two 2-day trials in a random order. On day 1, the participants underwent five 12 min bouts of cycling exercise with a bout of higher intensity exercise (4 min) after each and then a bout of lower intensity cycling (2 min). The total exercise time was 90 min. After the exercise, the participants ingested three high-fat or low-fat meals. On Day 2, the participants were asked to rest in the laboratory and ingest a high-fat meal. Their postprandial reaction after a high-fat meal was observed.

**Results:**

Postprandial triglyceride concentrations in the high-fat diet trial and low-fat diet trial exhibited nonsignificant differences. Total TG AUC were no significantly different on HF trial and LF trial (HF: 6.63 ± 3.2; LF: 7.20 ± 3.4 mmol/L*4 h. *p* = 0.586). However, the postprandial fat oxidation rate total AUC (HF: 0.58 ± 0.1; LF: 0.39 ± 0.2 g/min*4 h. *p* = 0.045), plasma glucose, and insulin concentration of the high-fat trial were significantly higher than those of the low-fat trial.

**Conclusions:**

This study revealed that meals with distinct nutritional contents after a 90-min exercise increased the postprandial fat oxidation rate but did not influence the postprandial lipemia after a high-fat meal the next day.

## Introduction

Elevated postprandial triglyceride (TG) concentrations have been suggested to significantly increase the risk of metabolic disease [1]. A single session of exercise can decrease postprandial TG concentrations the next day [2, 3]. Numerous studies have found that energy expenditure during exercise may play a vital role in postprandial TG response [4, 5]. Exercise decreases postprandial lipemia the next day by enhancing the lipoprotein lipase (LPL) activity [6], increasing the postprandial fat oxidation rate [7], and improving the insulin sensitivity after exercise [8]. However, the exact mechanism underlying this phenomenon remains unknown.

Diverse types of meals with a varying nutritional content may influence postprandial TG concentration. Under nonexercise conditions, high-carbohydrate diets have been suggested to decrease the hepatic fatty acid oxidation and increase plasma TG concentration [9]. After exercise, high-carbohydrate diets induce a higher postprandial TG concentration compared with low-carbohydrate diets [10]. This may be because high-carbohydrate diets decrease postprandial fat oxidation [10]. However, high-fat (HF) postexercise meals have also been found to increase postprandial fat oxidation [11]. The relationship between a diet’s varying nutritional content and postprandial fat oxidation remains unclear.

Postprandial fat oxidation may play a major role in postprandial lipemia. High-intensity interval exercise may increase postprandial fat oxidation and reduce postprandial TG concentration the next day [12, 13]. In addition, HF postexercise meals increased postprandial fat oxidation [11]. The effect of a higher postprandial fat oxidation rate induced by HF meals after exercise on postprandial TG concentration remains unclear. The objective of this study was to investigate the effects of ingesting HF or low-fat (LF) meals with the same calorie intake after exercise on postprandial TG concentration and postprandial fat oxidation based on an oral fat tolerance test (OFTT) the next day.

## Methods

### Participants

Eight healthy male participants were recruited (age 22 ± 1.3 yr, height 170.1 ± 4.7 cm, weight 75.4 ± 17.5 kg; Table 1). No participant received professional exercise training, but had the habit of exercising two to three times a week. The participants did not present any metabolic disorders, lipemia, or other problems rendering them unfit to engage in exercise. A questionnaire was used to screen for physical activity level and any potential health issues before testing. After completely understanding the experiment, the participants signed an informed consent form. This study was approved by the Institutional Review Board of Changhua Christian Hospital (CCH IRB No 151221) in Taiwan.

**Table 1:** The participants physiological information and fasting plasma biochemistry

	LF	HF	*p* value
Body Mass Index (BMI, kg/m^2^)	24.1 ± 5.6		
Body fat (%)	21.0 ± 4.7		
Average heart rate during exercise (beat/min)	147.15 ± 10.55	148.38 ± 8.19	0.414
Energy expenditure during exercise (Kcal)	987.4 ± 142.4	997.8 ± 129.2	0.527
TG (mmol/L)	0.93 ± 0.48	0.62 ± 0.22	0.067
Glucose (mmol/L)	4.86 ± 0.49	5.01 ± 0.44	0.505
Insulin (pmol/L)	1.12 ± 0.47	1.58 ± 0.90	0.077
NEFA (mmol/L)	0.85 ± 0.19	0.80 ± 0.16	0.597
Glycerol (μ mol/L)	46.57 ± 21.80	47.75 ± 14.13	0.791

Values are mean SD, *n* = 8. LF, low fat diet trial; HF, high fat diet trial; TG, triglyceride; Non-esterified fatty acids

### Design

A crossover design approach was adopted in this study. The experiment involved two trials, namely an LF diet trial and an HF diet trial. Participants first underwent a pretest to measure their VO_2max_ and calculate the intensity of their interval training during the formal experiment. The pretest and formal experiment had to take place at least 7 days apart. The participants exercised at 66% VO_2max_ for 90 min in the morning on the first day of the formal experiment. Interval training was incorporated five times during the process, and at the end of the exercise, three LF or HF meals with equal calorie intakes were administered. The experimental sequences occurred in a random order, with each test conducted at least 7 days apart from the others to avoid influences.

### Protocol

#### Pretest

The pretest in this study involved using stationary bicycles to measure VO_2max_ and assess exercise intensity. Participants arrived at the laboratory in the afternoon and were asked to wear a heart rate monitor wristband (Polar Electro, Kempele, Finland) and a precalibrated breath-by-breath gas analyzer (Cortex, Metamax 3B, Leipzig, Germany), which were used to collect relevant measurements during the exercise. First, participants’ gas samples during the resting state (sitting) were collected for 5 min to determine their energy expenditure at the resting state. Subsequently, a VO_2max_ test was conducted at a fixed cadence and during an incremental amount of pedal power (in W) on a cycle ergometer. Specifically, cadence was maintained at 70 to 80 rpm under an intensity of 75 W, while the power output was incremented by 25 W every 3 min until the participant was exhausted. During the test period, the oxygen amount, partial pressure of oxygen (PO_2_), partial pressure of carbon dioxide (PCO_2_), energy expenditure, and heart rate were recorded at each stage to calculate the amount of energy expended at 66% VO_2max_ and the usage of carbohydrate and fat. The fat and carbohydrate oxidation rates were calculated using the following formula [14]:

$$ \mathrm{Fat}\ \mathrm{oxidation}\ \left(\mathrm{g}/\min \right)=1.695\times {\mathrm{VO}}_2-1.701\times {\mathrm{VCO}}_2. $$

$$ \mathrm{Carbohydrate}\ \mathrm{oxidation}\ \left(\mathrm{g}/\min \right)=4.585\times {\mathrm{VCO}}_2-3.226\times {\mathrm{VO}}_2. $$

### Formal experiment

The experiment was conducted over 2 days. Four days before the first formal experiment, a nutritionist individually provided all of the participants with diet-related knowledge and asked them to avoid ingesting an excessive amount of fat and calories as well as alcohol and caffeine. To facilitate dietary control, the participants were asked to record the meals they had ingested during the 3 days preceding the formal experiment and to ingest the same meals 3 days before the subsequent formal experiment. All participants were also asked to avoid excessive physical activities and heavy training 3 days before the formal experiment. Participants arrived at the laboratory between 08:00 and 09:00 in the morning on the first day of the formal experiment. They rested for 10 min before putting on a polar watch and gas analyzer to determine the actual exercise intensity. First, participants rode a cycle ergometer for 12 min at 66% VO_2max_, after which the intensity was increased to 85% VO_2max_ for 4 min and then decreased to 50% VO_2max_ for 2 min. Completing these three intensities was considered a cycle, and there was in total five cycles. During the exercise, 200 mL of drinking water was provided to the participants every 20 min to prevent dehydration.

At the end of the exercise, an LF or HF meal was administered to the participants from 09:45–10:45, at 12:30, and at 19:00. All meals were prepared by a nutritionist. In the HF trial, the meals had a total calorie intake of 2437.7 kcal and included breakfast (full-cream milk, peanut butter toast, and 8 g of nuts), lunch (bubble tea, creamy bacon pasta, and kiwi), and dinner (110 g of KFC Chizza and a KFC Zinger). The amounts of fat, protein, and carbohydrate in the three meals were 44% (119.7 g), 12% (71.9 g), and 44% (268.2 g) of the total calorie intake, respectively. In the LF trial, the meals had a total calorie intake of 2448.2 kcal and included breakfast (40 g of whey protein, kiwi, banana, Laba congee, and lemon tea), lunch (40 g of whey protein, 200 g of white rice, 150 g of sweet mung bean soup, and kiwi), and dinner (40 g of whey protein, boiled vegetables, 200 g of white rice, a tea egg, black tea, and banana). The amounts of fat, protein, and carbohydrate in the three meals were 6% (15 g), 20% (126.3 g), and 74% (452 g) of the total calorie intake, respectively. The macronutrient consumption for LF and HF were listed in Table 2.

**Table 2:** The macronutrient consumption for LF and HF

	Carbohydrate (g)	Protein (g)	Fat (g)	Total calorie (Kcal)
HF				
Breakfast	41	17.6	33.5	535.9
Lunch	141.1	15.1	50.4	1078.4
Dinner	86.1	39.2	35.8	823.6
Total	268.2 (44%)	71.9 (12%)	119.7 (44%)	2437.9
LF				
Breakfast	157	31.3	3.5	784.7
Lunch	136	51	6.5	806.5
Dinner	159	44	5	857
Total	452 (74%)	126.3 (20%)	15 (6%)	2448.2

*LF* Low fat diet trial, *HF* High fat diet trial

The participants returned to the laboratory at approximately 08:00 AM on the second day of the formal experiment to undertake an OFTT in the fasting state. After 10 min of rest, the participants’ fasting blood samples were collected through venipuncture. Subsequently, the participants were given a fixed HF meal and rested in the laboratory for 4 h. Further blood samples were collected at 0.5, 1, 2, 3, and 4 h after the end of the meal. Postprandial gaseous samples were collected by a precalibrated breath-by-breath gas analyzer (Cortex, Metamax 3B, Leipzig, Germany) from the resting sitting position for 5 min at each time point to calculate the participants’ postprandial fat oxidation rate.

### Blood sample collection

In the experiment, 10-mL blood samples were collected using an intravenous catheter (Venflon 20G cannula, Sweden) and three-way connector (Connecta Ltd., Sweden). Samples were collected 30 min before and immediately and 1, 2, 3, and 4 h after a meal. The blood samples were collected into collection Vacutainers containing ethylenediaminetetraacetic acid (EDTA). To prevent the blood from clotting in the catheter, we used 10 mL of isotonic saline to clean the catheter. The Vacutainers were centrifuged for 20 min at 2000×*g* at 4 °C. Blood plasma was extracted and stored at − 80 °C for subsequent biochemical analysis.

The plasma concentrations of TG, glucose (GLU), glycerol (GLY), and non-esterified fatty acids (NEFA) were determined using an automatic biochemistry analyzer (Hitachi 7020, Tokyo, Japan) and commercially available reagents (GOD-PAP method, Randox, Ireland). The inter-assay and intra-assay CVs were: TG (1.9% & 0.6%, respectively); GLU (2.2% & 3.7%, respectively); GLY (0.9% & 6.4%, respectively); NEFA (2.6% & 4.4%, respectively). The plasma concentrations of insulin was determined using an automatic biochemistry analyzer (Elecsys 2010, New York, USA) and commercially available reagents (Electrochemiluminescence immunoassay method, Roche, Switzerland). The inter-assay and intra-assay CVs were 0.83 and 2.6%, respectively.

### Oral fat tolerance test (OFTT)

All the meals provided for the OFTT were designed by a nutritionist and have been used in a previous study [7, 15]. The meals were composed of toast, butter, cheese, muesli, and fresh cream. The meals provided 1.2 g of fat per kg body weight, 1.1 g of carbohydrate, 0.33 g of protein, and 16.5 kcal of energy. The nutritional contents of the meals were obtained from the packaging labels. During the experiment, the participants were required to ingest their OFTT meals within 15 min.

### Statistical analysis

All data were presented as mean ± standard deviation. The t-test was used to test the concentration difference in the area under the curve (AUC) of each dependent variable between the two groups. Two-way ANOVA with repeated measures was performed to analyze the difference in blood biochemical values between the groups and at various time points. A statistically significant difference required posthoc comparison using the Bonferroni method. Significance was defined as α = 0.05. The G*Power 3 software program was used to calculate the sufficient sample size with an α value of 5% and a power of 0.8.The sufficient sample size obtained was eight participants.

## Result

The participants physiological information and fasting plasma biochemistry.

There were no significantly different between HF and LF in the average heart rate (*p* = 0.414) and energy expenditure (*p* = 0.527) during exercie. The fasting concentrations from the plasma biochemistry did not differ on the morning of Day 2 in all trials (Table 1).

### TG concentrations, fat oxidation and carbohydrate oxidation

**Fig. 1:**
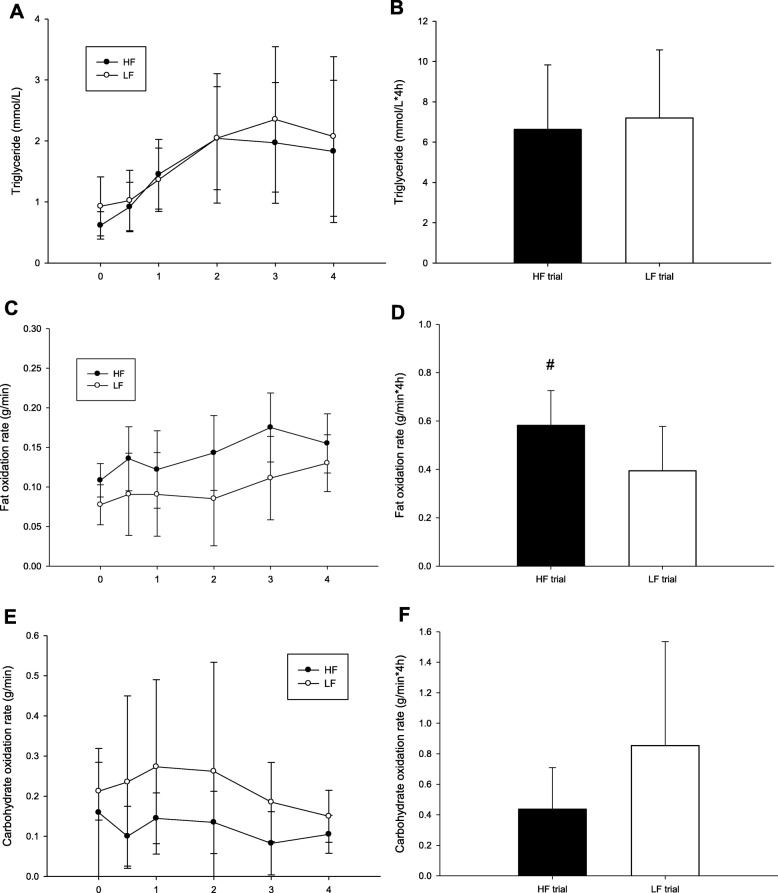
The postprandial TG concentrations over the 4 h (**a**), the TG area under the curve in 4 h (**b**), the fat oxidation rate over the 4 h (**c**) and the fat oxidation rate area under the curve in 4 h (**d**). ^#^ HF was significantly higher than those for the LF

There were no differences between HF and LF in TG concentrations (trial × time, *p* = 0.219; trial, *p* = 0.501; time, *p* < 0.001; Fig. 1a), TG AUC (*p* = 0.586; Fig. 1b), and fat oxidation rate (trial × time, *p* = 0.474; trial, *p* = 0.086; time, *p* = 0.001; Fig. 1c). Figure 1d demonstrates the fat oxidation rate AUC in the HF trial was significantly higher than that in the LF trial (*p* = 0.045). There were no differences between HF and LF in the carbohydrate oxidation rate (trial × time, *p* = 0.479; trial, *p* = 0.387; time, *p* = 0.239; Fig. 1e) and the AUC of carbohydrate oxidation rate (*p* = 0.216; Fig. 1f).

### GLU and insulin

**Fig. 2:**
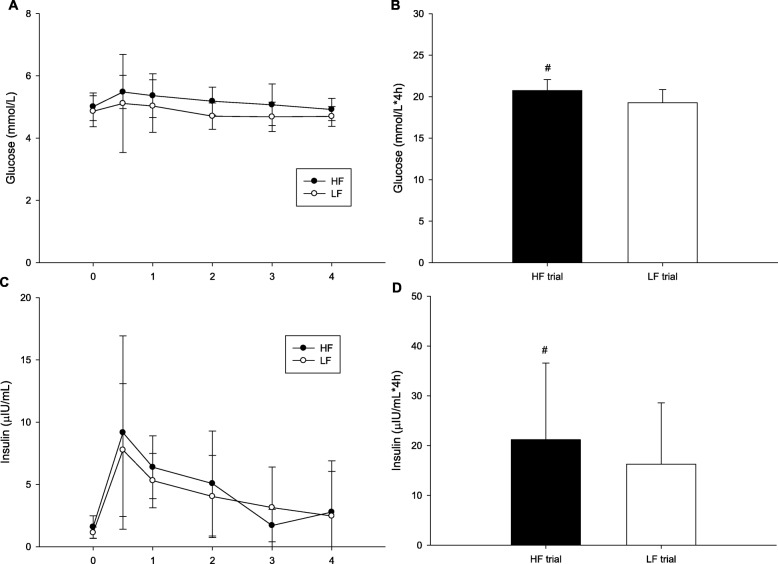
The postprandial glucose concentrations over the 4 h (**a**), the glucose area under the curve in 4 h (**b**), the insulin over the 4 h (**c**) and the insulin area under the curve in 4 h (**d**). ^#^ mean HF was significantly higher than those for the LF

Plasma GLU concentrations exhibited no significant differences between trials (trial × time, *p* = 0.822; trial, *p* = 0.021; time, *p* = 0.321; Fig. 2a). Figure 2b indicates that the plasma GLU AUC was higher in the HF trial than in the LF trial (*p* = 0.007). There were no differences between HF and LF in the concentrations of insulin (trial × time, *p* = 0.503; trial, *p* = 0.284; time, *p* < 0.001; Fig. 2c), but the plasma insulin AUC was higher in the HF trial than in the LF trial (*p* = 0.015; Fig. 2d).

### NEFA and GLY

**Fig. 3:**
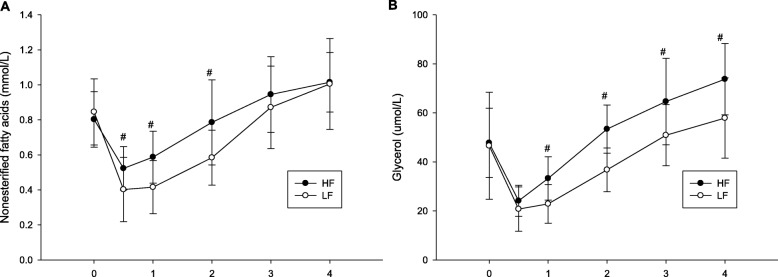
The postprandial nonesterified fatty acids concentrations over the 4 h (**a**) and glycerol concentrations over the 4 h (**b**). ^#^ mean HF was significantly higher than those for the LF

Plasma nonesterified fatty acids (NEFA) concentrations has significant interaction (trial × time, *p* = 0.042; Fig. 3a). At 0.5, 1 and 2 h after the meal in the HF trial were significantly higher than the LF trial (0.5 h, *p* = 0.022; 1 h, *p* = 0.005; 2 h, *p* = 0.012). Plasma glycerol (GLY) concentrations has significant interaction (trial × time, *p* = 0.038; Fig. 3b). At 1, 2, 3 and 4 h after the meal in the HF trial were significantly higher than the LF trial (1 h, *p* < 0.001; 2 h, *p* < 0.001; 3 h, *p* = 0.005; 4 h, *p* = 0.007).

## Discussion

He present study revealed that among exercise interventions with different intensities and the same energy expenditure, HIIE is more capable of reducing the postprandial TG concentrations.

This study revealed that various contents in meals after a 90-min exercise significantly raised the fat oxidation rate after an HF meal the next day, but it did not affect the plasma TG concentration. In addition, the results demonstrated that ingesting an HF meal after exercise significantly increased postprandial GLU and insulin concentrations.

This study revealed that when the same amount of energy expended during exercise and the same calorie intake on the previous day, meals with dissimilar fat contents did not influence the postprandial TG concentration the next day. In a previous study, low-carbohydrate diets increased the postprandial fat oxidation and decreased the postprandial TG concentration compared with high-carbohydrate diets [10]. However, the fat content in the low-carbohydrate diet trial was 72.2% in this study. Eating high-fat-content meals in the daily life is difficult. Therefore, we decreased the fat content to 44% in the meals of the HF trial and successfully increased the postprandial fat oxidation compared with the LF trial, but there were no differences in the postprandial TG concentration between the HF and LF trial. The higher concentration of insulin observed in the HF trial may play a role in the absence of change in the postprandial TG concentration.

The higher insulin concentration in the postprandial period may decrease the LPL activity and influence the postprandial TG response. Previous findings have suggested that ingesting HF meals results in reduced insulin sensitivity [16–18]. Bachmann et al. (2001) fed 12 participants HF and LF meals for 3 days in a row and assessed their insulin sensitivity. The results indicated that the insulin sensitivity fell below 83.3 ± 5.6% of the baseline, and insulin sensitivity after an LF diet exhibited a nonsignificant difference [19]. Although we did not calculate the insulin sensitivity in this study, our results demonstrated that the GLU and insulin concentrations of the HF group were considerably higher than those of the LF group, indicating that the HF group was less sensitive to insulin. Based on other data from the present study, the postprandial NEFA and GLY concentrations were higher in the HF trial compared with the LF trial. This may reflect a reduction of the insulin sensitivity in the HF trial compared with the LF trial. A higher insulin concentration and lower insulin sensitivity have been suggested to decrease the LPL activity and clearance of TG from the blood circulation [20]. Therefore, a higher postprandial insulin response may reduce the positive effect of higher postprandial fat oxidation on postprandial TG concentration.

This study also revealed that the fat oxidation rate significantly increased in the HF trial. In previous studies on the effects of exercise interventions on postprandial lipemia, high-intensity interval training a day before OFTT was found to significantly increase the postprandial fat oxidation rate after an HF meal the next day, and the postprandial TG concentration was also considerably reduced after an OFTT [7]. These findings indicate that an increase in the postprandial fat oxidation rate may influence the postprandial TG concentration. In addition to high-intensity interval training, ingesting HF meals was similarly suggested to elevate the postprandial fat oxidation rate [10, 11]. However, no studies have investigated whether an increase in fat oxidation rate due to HF meals influences TG concentrations after an HF meal. Although this study revealed an increase in postprandial fat oxidation rate, the postprandial TG concentration was not affected.

The primary limitation of this study is that a control trial (no exercise group) was not used. It is difficult to determine whether the postprandial TG concentration was or not affected in the exercise trial. However, the objective of this study was to investigate the effects of ingesting HF or LF meals on postprandial TG concentration and postprandial fat oxidation after an OFTT the next day. Therefore, a control trial did not appear to be critical for this study. The second limitation of this study was the difference in the protein content among trials. The acute effect of the ingestion of additional protein into an HF meal may reduce the postprandial TG concentration [21, 22]. However, no study has investigated the long-term effect of protein ingestion or the effect of protein on the day before the HF meal test. We believe a higher content of protein the day before the HF meal did not influence the results in this study.

## Conclusion

This study revealed that various contents in meals after a 90-min exercise did not influence the postprandial lipemia after an OFTT the next day. Compared with LF meals, HF meals resulted in a higher fat oxidation rate, GLU level, and insulin concentration after an OFTT. Thus, HF diets can cause a reduction in insulin sensitivity. Nevertheless, future studies should consider using the OGTT method to investigate the effects of various meals after exercise on insulin sensitivity.

## Data Availability

The data analyzed during the present study are available from the corresponding author on reasonable request.
